# Microbial Diversity and Antibiotic Susceptibility Pattern of Bacteria Associated with Motorcycle Helmets

**DOI:** 10.1155/2020/8877200

**Published:** 2020-10-29

**Authors:** Sanjeep Sapkota, Sujan Khadka, Sanjib Adhikari, Ashish Parajuli, Hemraj Kandel, Ramesh Sharma Regmi

**Affiliations:** ^1^Department of Microbiology, Birendra Multiple Campus, Tribhuvan University, Bharatpur, Chitwan, Nepal; ^2^State Key Laboratory of Respiratory Disease, Guangzhou Institutes of Biomedicine and Health, Chinese Academy of Sciences, Guangzhou, China; ^3^University of Chinese Academy of Sciences, Beijing, China; ^4^State Key Laboratory of Environmental Aquatic Chemistry, Research Center for Eco-Environmental Sciences, Chinese Academy of Sciences, Beijing, China; ^5^Department of Microbiology, St. Xavier's College, Kathmandu, Nepal; ^6^Department of Biotechnology, National College, Kathmandu, Nepal

## Abstract

**Background:**

Motorcycle helmets can serve as a potential vehicle for the transmission of pathogenic bacteria and fungi with serious health implications. The main aim of this study was to explore the microbial diversity associated with the motorcycle helmets and determine the antibiotic susceptibility profile of the bacterial isolates.

**Methods:**

A descriptive cross-sectional study was carried out among the teaching staffs of Birendra Multiple Campus, Bharatpur, Nepal. A total of 130 motorcycle helmets worn by the teaching staffs of the Birendra Multiple Campus, Bharatpur, were included in the study for microbiological investigations.

**Results:**

Of the total 130 motorcycle helmets analyzed, 392 bacteria and 346 fungi belonging to seven different genera were recovered. *Staphylococcus aureus* 89 (22.7%) was the predominant bacteria followed by *S. epidermidis* 77 (19.6%) and *E. coli* 54 (13.8%), whereas *Aspergillus niger* 67 (19.4%) was the predominant fungi followed by *A. fumigatus* 49 (14.2%). Antibiotic susceptibility test was performed by the disc diffusion method for all the bacterial isolates. Tetracycline, gentamycin, and cotrimoxazole were the most effective antibiotics for Gram-positive isolates, whereas Gram-negative isolates were sensitive towards imipenem and ciprofloxacin. Of the total bacterial isolates, 153 (39.0%) were multidrug-resistant (MDR), 10.4% were extended-spectrum beta-lactamase (ESBL) producers, and 4.3% were metallo-beta-lactamase (MBL) producers and, out of 89 isolates of *Staphylococcus aureus,* 30 (33.7%) were detected as methicillin-resistant *Staphylococcus aureus* (MRSA).

**Conclusion:**

The findings suggest that motorcycle riders should follow good hygiene practices and regularly clean their helmets with suitable sterilants to avoid the risk of microbial contamination and reduce the associated risks.

## 1. Introduction

Transportation is essential to every human community and one of the easier means of transportation in most part of the world is the motorcycle. Motorcycles are fashionable means of transportation especially for short distances. Its frequent use and patronage is very high since it is quicker and convenient and can easily maneuver through the regular traffic jam and get to any destination, provided there is road facility [1]. Motorcycle helmets (MCHs) protect motorcyclists from serious head and brain injuries in the event of an accident [2]. However, constant handling and use of MCHs by different individuals could create a prime breeding ground for many microorganisms such as bacteria and mildews [3] and there is a possible transmission of pathogenic microorganisms as well as communicable diseases among users.

Human skin, the human body's largest organ, is constantly in contact with environmental microorganisms and become readily colonized by certain microbial species. The adult human skin can hold about 10^12^ cfu/ml bacteria [4]. The colonization of potentially pathogenic organisms on inanimate objects like mobile phones, stethoscopes, currency notes, and computer keyboards has been reported as a potential vehicle for transmission of pathogenic organisms and important source of infections [5–8]. Some previous studies have revealed the presence of *Staphylococcus aureus*, *Enterobacter aerogenes*, *Pseudomonas aeruginosa*, *Staphylococcus epidermidis*, *Escherichia coli, Bacillus* spp*., Aspergillus* spp.*, Candida* spp.*, Rhizopus* spp., and *Penicillium* spp. from motorcycle helmets [9, 10]. These normal microbiota can produce diseased condition if introduced into foreign locations or compromised hosts [11].

Antibiotics are a crucial line of defense against bacterial infections; however, bacteria resisting to antibiotics is a major public health concern [12, 13]. Bacteria can be intrinsically resistant or acquire resistance to antibiotics through horizontal gene transfer of resistant genetic mobile elements and alteration in genetic composition [14]. There are increasing evidences of multidrug-resistant MRSA and ESBL producing bacterial isolates in inanimate objects [8, 15, 16]. In Nepal, there is paucity of information on potential hazards associated with common use of motorcycle helmets by different people. In context of country like ours, we do not find any research works conducted to reveal microbes that contaminate motorcycle helmets. A very few research works have been conducted even in the world regarding microbial contamination of motorcycle helmets. Since most of the organisms that contaminate the motorcycle helmets are capable of causing infections, this work certainly has health implications. Similarly, at the time when antibiotic resistance among nonclinical bacterial isolates is also increasing, understanding the AST pattern of the bacteria recovered from motorcycle helmets will be helpful in combating the battle with antimicrobial resistance.

## 2. Methods

### 2.1. Study Site

A descriptive cross-sectional study was carried out among the teaching staffs of Birendra Multiple Campus, Bharatpur, Nepal. Birendra Multiple Campus is a government-funded constituent campus of Tribhuvan University situated in Province No. 3. The campus has around 3000 students and 300 teaching and nonteaching staffs and more than 95% of the staffs in the campus rely on motorcycle as their daily mode of conveyance. A total of 130 teaching staffs were recruited in the study by random sampling method. Laboratory investigations were carried out in the Microbiology Laboratory of the campus. Data related to sociodemographic characteristics and personal hygiene practices were collected through interview method.

### 2.2. Collection and Processing of the Sample

The samples were collected from motorcycle helmets using two sterile swabs according to the method described by Sepehri et al. [16]. Briefly, two sterile swabs moistened with sterile water were rotated over the inner surface of the helmets. The first swab was immediately streaked onto nutrient agar (NA) plates and MacConkey (MAC) agar plates, whereas the second swab was streaked on Potato Dextrose Agar (PDA) supplemented with chloramphenicol. NA and MAC agar plates were incubated aerobically at 37°C for 24 h, while PDA plates were incubated at 28°C for 5–7 days. Distinct colonies were subcultured on NA until pure cultures were obtained.

### 2.3. Identification of Bacteria and Fungi

Identification of bacterial isolates was carried out based on their cultural, morphological, and biochemical characteristics [17]. Fungal mycelium was scraped off with the help of inoculating needle and teased out in a drop of lactophenol cotton blue on a clean and grease-free slide as described by Samson and Van Reenen-Hoekstra [18] and examined microscopically. Fungi were differentiated on the basis of cultural and morphological characteristics as reported by Samson et al. [19].

### 2.4. Antibiotic Susceptibility Test (AST) of the Bacterial Isolates

Antibiotic susceptibility tests were performed on Mueller-Hinton Agar (MHA) following Clinical and Laboratory Standards Institute (CLSI) guidelines [20]. The turbidity of the inoculums was adjusted to the equivalent turbidity of 0.5 McFarland standards. Eighteen-hour cultures of test organisms incubated at 37°C were standardized by diluting to 0.5 McFarland turbidity standards before spreading over the surface of Mueller–Hinton agar (MHA) and allowed to dry for 2–5 minutes. Using sterile tweezers, antimicrobial discs such as tetracycline (30 *μ*g), cloxacillin (5 *μ*g), erythromycin (15 *μ*g), amikacin (30 *μ*g), ciprofloxacin (5 *μ*g), cotrimoxazole (25 *μ*g), gentamycin (10 *μ*g), azithromycin (15 *μ*g), cefoxitin (30 *μ*g), and vancomycin (30 *μ*g) for Gram positive bacteria, and, for Gram negative bacteria, chloramphenicol (30 *μ*g), imipenem (10 *μ*g), amikacin (30 *μ*g), ciprofloxacin (5 *μ*g), azithromycin (15 *μ*g), nitrofurantoin (300 *μ*g), tetracycline (30 *μ*g), ceftazidime (30 *μ*g), gentamycin (10 *μ*g), and cotrimoxazole (25 *μ*g) were placed widely spaced aseptically on the surface of MHA plate. Tweezers were reflamed after application of each disc. The plates were then incubated at 37°C for 24 h. Following incubation, diameter of inhibition zone (DIZ) was measured with a transparent ruler and the results were interpreted as resistant, intermediate, and sensitive according to the Clinical and Laboratory Standards Institute guidelines [20]. Multidrug-resistant (MDR) strain was identified as resistant to ≥3 antimicrobial classes [21].

### 2.5. Screening of MRSA, ESBL, and MBL Producers

Cefoxitin (30 *μ*g) disc was used for screening of MRSA. Isolates showing inhibition zone ≤21 mm around cefoxitin disc were identified as MRSA strain.

Screening of ESBL producers was performed by using ceftazidime (30 *μ*g) and cefotaxime (30 *μ*g) discs. The zone of inhibition (ZOI) ≤22 mm for ceftazidime and ≤27 mm for cefotaxime was considered as a potential ESBL producer as recommended by Clinical and Laboratory Standards Institute (CLSI). Possible ESBL producers were subjected to combined disc test for phenotypic confirmation as recommended by CLSI [20]. The combination of ceftazidime and cefotaxime alone and in combination with clavulanic acid (CA) (10 *μ*g) was used for the confirmation of ESBL producing isolates. An increased ZOI of ≥5 mm for either antimicrobial agent tested in combination with CA versus its zone when tested alone was interpreted as positive for ESBL production.

Imipenem resistant Gram-negative isolates were selected for the detection of MBL production by disc potentiation method as previously described [22, 23]. Briefly, bacterial isolates (adjusted to 0.5 McFarland turbidity standards) were aseptically swabbed on Mueller–Hinton (MH) agar plates and standard antibiotic disks of imipenem (10 *μ*g) and meropenem (10 *μ*g) impregnated with EDTA (1 *μ*g) were aseptically placed on MH agar plates. Supplementary imipenem (10 *μ*g) and meropenem (10 *μ*g) disks without EDTA were also placed alongside the antibiotic disks impregnated with the chelating agent (EDTA) at a distance of 20 mm apart. All plates were then incubated at 30°C for 18–24 h and zone of inhibition were recorded after incubation. A difference of ≥7 mm between the zones of inhibition of any of the carbapenem disks with or without the chelating agents was considered as metallo *β*-lactamase production [23].

### 2.6. Quality Control

Reagents and media were frequently monitored for their expiry date and proper storage conditions. For the standardization of Kirby–Bauer test and performance testing of antibiotics and MHA, control strains of *E. coli* (ATCC 25922) and *S. aureus* (ATCC 25923) were used. Quality of sensitivity tests was maintained by maintaining the thickness of MHA at 4 mm and the pH at 7.2–7.4.

### 2.7. Data Management and Statistical Analysis

All the data obtained were analyzed using Statistical Package for Social Sciences (SPSS) software (Version 20.0). Chi-square test was used to determine the association between some selected variables and a *p* value ≤0.05 was considered to have significant association.

## 3. Results

### 3.1. Growth Pattern of Isolates

In the study, altogether 130 motorcycle helmets were analyzed, from which 392 bacteria and 346 fungi belonging to seven different genera were recovered. Out of them, 229 (58.0%) were Gram positive bacteria belonging to three genera, *Staphylococcus, Bacillus,* and *Micrococcus,* whereas 163 (42.0%) were Gram negative bacteria belonging to five genera, namely, *Pseudomonas, E. coli, Enterobacter, Salmonella,* and *Klebsiella*. *Staphylococcus aureus* 89 (22.7%) was the predominant flora in Gram positive category, followed by *S. epidermidis* 77 (19.6%), *Bacillus* spp. 44 (11.2%), and *Micrococcus* spp. 19 (4.8%), whereas, in Gram negative category, *E. coli* 54 (13.8%) was the predominant flora followed by *Enterobacter* spp. 38 (9.7%), *Klebsiella* spp. 33 (8.4%), and equal number of *Pseudomonas aeruginosa* and *Salmonella* spp. 19 (4.8%) (Table 1).

Altogether, 346 fungi belonging to seven different genera were isolated from motorcycle helmets. Out of 346 fungi isolated, 20 (5.8%) were yeast belonging to only one genus which was *Candida albicans,* whereas 326 (94.2%) were molds belonging to 6 different genera such as *Aspergillus, Rhizopus, Mucor, Alternaria, Fusarium, and Penicillium.* Three species of *Aspergillus* were identified and they were *A. niger, A. fumigatus,* and *A. flavus. Aspergillus niger* 67(19.4%) was the most predominant mold followed by *A. fumigatus* 49 (14.2%). In contrast, *Alternaria* spp. 19 (5.5%) were the least prevalent mold observed in the study (Table 2).

### 3.2. Distribution of Microbial Growth Based on Different Variables

Among 130 helmet samples, 115 were from males and 15 were from females. Helmet worn by male showed 73.9% bacterial and 65.2% fungal growth, while 80.0% bacterial and 66.7% fungal growth were recovered from the helmet worn by females. Helmets that were worn by the 40–60 age grouped people harbored more bacterial (77.8%) and fungal (82.5%) growth. Presence of microorganism in helmet was not associated with the gender and age of the rider (*p* > 0.05). Helmets that were worn for more than 3 years showed higher bacterial (89.1%) and fungal (80.4%) growth than helmets used for less than 3 years (*p* < 0.05). Helmets which were never washed or disinfected showed higher bacterial (86.8%) and fungal (83.3%) growth as compared to those helmet frequently washed or disinfected (*p* < 0.05). Shared helmet seemed to be more hazardous as 79.2% bacterial and 71.88% of fungal growth was recorded (*p* < 0.05) (Table 3).

### 3.3. Antibiotic Susceptibility Pattern of Gram-Positive Isolates

*In vitro* drug susceptibility was performed for all Gram positive isolates by Modified Kirby Bauer Disc Diffusion method. Tetracycline, gentamicin, and cotrimoxazole were the most effective antibiotics for Gram positive isolates. *Staphylococcus epidermidis* showed 100% sensitivity towards gentamicin. High level of resistance was shown by the *Bacillus* spp. towards cloxacillin (59.1%), erythromycin (65.9%), and amikacin (52.3%). On the other hand, *Micrococcus* spp. showed 63.2% and 52.6% resistance towards erythromycin and cefoxitin, respectively. Among 89 isolates of *Staphylococcus aureus*, 30 (33.7%) were detected as MRSA (Table 4).

### 3.4. Antibiotic Susceptibility Pattern of Gram-Negative Isolates

Gram negative isolates were sensitive towards imipenem and ciprofloxacin. *Klebsiella* spp. and *Salmonella* spp. were 100.0% sensitive to gentamicin and imipenem, respectively. The majority of Gram negative isolates showed high rate of resistance to cotrimoxazole and tetracycline. High level of resistance was shown by *Pseudomonas aeruginosa* towards nitrofurantoin (57.9%), tetracycline (63.2%), and gentamicin (73.7%). Among 392 bacterial isolates, 153 (39.0%) were multidrug-resistant (Table 5).

### 3.5. Frequency of ESBL and MBL Producers

All Gram negative isolates were further tested for ESBL and MBL activity. The overall percentage for ESBL and MBL producers was 10.4% and 4.3%, respectively. High prevalence of ESBL producers was seen among *Pseudomonas aeruginosa* (21.0%) and *Klebsiella* spp. (15.1%), whereas none of the *Enterobacter aerogenes* isolates was detected as ESBL producers. *Pseudomonas aeruginosa* (10.5%), *Klebsiella* spp. (6.1%), and *E. coli* (5.6%) were detected as MBL producers (Table 6).

## 4. Discussion

Microbiology standards in hygiene are prerequisite for a health living. It is not uncommon however to observe shift in hygienic practices that deviate from standards in developing and developed countries. Motorcycle helmets have been identified as a possible vehicle for the transmission of pathogenic bacteria and fungi; so, efforts to minimize transfer of such agents through good hygiene practice (GHP) are essential to maintain good health [10].

In this study, the number of bacteria isolated (392) was higher than the number of fungi (346). The variety of bacterial and fungal genera obtained in the study is similar to the works done previously in Lagos metropolis, Nigeria [9, 10, 24]. Additionally, *Micrococcus* spp. was isolated in the present study. Similarly, apart from the work in Nigeria, two new genera of the fungi *Mucor* and *Alternaria* were isolated in this study. *Staphylococcus aureus*, the predominant flora in our study, is a normal flora of skin but it is an opportunistic pathogen as it is known to cause boils, abscesses, wound infections, toxic shock syndrome, pneumonia, and other diseases [25, 26]. Similarly, *Staphylococcus epidermidis* is also a skin commensal flora but is also responsible for endocarditis and infections of patients with lowered immunity [27]. Their presence indicates that the use and sharing of motorcycle helmets can lead to transmission of serious skin infections. The presence of coliforms like *E. coli* and *Enterobacter aerogenes* indicates possible fecal contamination [28]. Its implication is that handling of motorcycle helmets could be a potential source of food poisoning when infected hands are used in eating and food preparation without proper hygiene of hand washing [26]. *Aspergillus fumigatus*, a common fungus in the environment, is responsible for allergies and has also been linked to severe asthma, sinusitis, and pulmonary aspergillosis [29]. Moreover, *Candida* species are the commensal flora of gastrointestinal tract, respiratory tract, and vagina and cause candidiasis and oral thrust [26].

A higher proportion of bacterial as well as fungal growth was reported from helmets worn by females and 40–60 age grouped riders (*p* > 0.05). This result is different from the study done in Islamic University of Gaza which reported the rate of incidence of contamination of mobile phones held by females was lower than that of male counterparts [30]. Usually, females keep longer nails which can directly transmit the microorganisms to the helmets [31]. Helmets that have been used for more than 3 years showed higher incidence of microbial growth. Helmets used for longer time period resulted in the attachment of microbes on the surface which results in the formation of biofilms and they are hard to remove [32]. Disinfected helmets showed the lower rate of microbial growth than that of nondisinfected helmets (*p* < 0.05). This result was in line with a study done in Nepal which showed the lower rate of microbial growth in disinfected mobiles phones [8]. Disinfection processes can actively kill the microbes from inanimate surfaces that lead to the reduction of microbial count [33, 34]. Moreover, shared helmet was found to have more bacterial as well as fungal growth than that of unshared one (*p* < 0.05). It is imperative that sharing of the inanimate objects facilitates the transfer of microbes from one surface to another. On microbial susceptibility testing, tetracycline, gentamicin and cotrimoxazole were the most effective antibiotics for Gram positive isolates, whereas Gram negative isolates showed sensitivity towards imipenem and ciprofloxacin. This was in contrary with the study done in Nigeria which showed bacterial sensitivity to oflaxacin was the highest [9]. The rate of antibiotic resistance is increasing rapidly and poses grave economical and medical implications such as high morbidity and mortality [35]. Majority of the *Salmonella* spp. and *Pseudomonas aeruginosa* were found to be resistant to gentamicin. Bacterial resistance to gentamicin has been earlier reported and the resistance evolution mechanism is attributable to mobile genetic elements [36]. The emergence of MRSA, ESBL, and MBL producing bacteria has become a serious concern. This study reported 33.7% of the *Staphylococcus aureus* as MRSA which was in tune with the previous study done in nonclinical samples [8, 33,37]. ESBLs are plasmid mediated TEM and SHV-type enzymes capable of hydrolysing penicillins, broad-spectrum cephalosporins, and monobactams [38]. Among 163 Gram negative isolates, 10.4% ESBL and 4.3% MBL producers were reported. In a study from Iran on mobile phones, ESBL was reported in 40% of the isolates. However, some fewer studies did not report a single case of ESBL and MBL producers [39, 40].

## 5. Conclusion

The current study shows that motor cycle helmets could serve as a potential vehicle for transmission of drug-resistant bacteria and fungi with serious health implications. Hence, good hygiene practice (GHP) should be followed by the motorcycle riders with regular cleaning of the helmets by suitable sterilants in order to reduce the occurrence of microbial contamination and transmission of microbes via helmets.

## Figures and Tables

**Table 1:** Growth pattern of bacteria.

SN	Name of organisms	Total cases (%)
1	*Staphylococcus aureus*	89(22.7)
2	*Staphylococcus epidermidis*	77(19.6)
3	*Bacillus* spp.	44(11.2)
4	*Micrococcus* spp.	19(4.8)
5	*Pseudomonas aeruginosa*	19(4.8)
6	*E. coli*	54(13.8)
7	*Enterobacter aerogenes*	38(9.7)
8	*Salmonella* spp.	19(4.8)
9	*Klebsiella* spp.	33(8.4)
	Total	392

**Table 2:** Growth pattern of fungi.

SN	Name of organisms	Total cases (%)
1	*Candida albicans*	20(5.8)
2	*Aspergillus niger*	67(19.4)
3	*A. fumigates*	49(14.2)
4	*A. flavus*	45(13.0)
5	*Rhizopus* spp.	55(15.9)
6	*Mucor* spp.	34(9.8)
7	*Alternaria* spp.	19(5.5)
8	*Fusarium* spp.	36(10.4)
9	*Penicillium* spp.	21(6.1)
	Total	346

**Table 3:** Distribution of microbial growth with different variables.

Serial number	Attributes		Sample size	Bacteria		Fungus	
				Growth *N* (%)	*p* value	Growth *N* (%)	*p* value
1	Gender	Male	115	85 (73.9%)	0.611	75 (65.2%)	0.912
		Female	15	12 (80.0%)		10 (66.7%)	

2	Age-group	20–40	28	19 (67.9%)	0.544	18 (64.3%)	0.861
		40–60	81	63 (77.8%)		52 (82.5%)	
		60+	21	15 (71.4%)		15 (71.4%)	

3	Length of helmet used	<1 yrs	25	15 (60.0%)	0.001^*∗*^	13 (52.0%)	0.023^*∗*^
		1–3 yrs	59	36 (61.1%)		35 (59.3%)	
		>3 yrs	46	41 (89.1%)		37 (83.4%)	

4	Hygienic practice	Disinfected	5	1 (20.0%)	0.001^*∗*^	1 (20.0%)	0.022^*∗*^
		Washed	42	24 (57.1%)		24 (57.1%)	
		None	83	72 (86.7%)		60 (83.3%)	

5	Sharing of helmet	Shared	96	76 (79.2%)	0.045^*∗*^	69 (71.9%)	0.008^*∗*^
		Not shared	34	21 (61.8%)		16 (47.0%)	

^*∗*^Significant at 5% level of significance.

**Table 4:** Antibiotic susceptibility pattern of Gram-positive isolates.

Serial number	Antibiotics used	Susceptibility pattern							
		*S. aureus* (*n* = 89)		*S. epidermidis* (*n* = 77)		*Bacillus* spp. (*n* = 44)		*Micrococcus* spp. (*n* = 19)	
		S (%)	R (%)	S (%)	R (%)	S (%)	R (%)	S (%)	R (%)
1	Tetracycline	88.8	11.2	85.7	14.3	88.6	11.4	84.2	15.8
2	Cloxacillin	91.0	9.0	89.9	10.1	40.9	59.1	73.7	26.3
3	Erythromycin	57.3	42.7	66.2	33.8	34.1	65.9	36.8	63.2
4	Gentamicin	94.4	5.6	100.0	0.0	84.1	15.9	78.9	21.1
5	Amikacin	82.0	18.0	85.7	14.3	44.7	52.3	68.4	31.6
6	Cotrimoxazole	89.9	10.1	92.2	7.8	63.6	36.4	63.2	36.8
7	Ciprofloxacin	77.5	22.5	90.9	9.1	93.2	6.8	68.4	31.6
8	Azithromycin	76.0	24.0	83.1	16.9	75.0	25.0	73.7	26.3
9	Cefoxitin	66.3	33.7	79.2	20.8	79.5	20.5	47.4	52.6
10	Vancomycin	88.8	11.2	88.3	11.7	65.9	34.1	63.2	36.8

*S* = sensitive; *R* = resistant.

**Table 5:** Antibiotic susceptibility pattern of Gram-negative isolates.

Serial number	Antibiotics used	Susceptibility pattern									
		*E. coli* (*n* = 54)		*Klebsiella* spp. (*n* = 33)		*P. aeruginosa* (*n* = 19)		*Salmonella* spp. (*n* = 19)		*Enterobacter aerogenes* (*n* = 38)	
		S (%)	R (%)	S (%)	R (%)	S (%)	R (%)	S (%)	R (%)	S (%)	R (%)
1	Chloramphenicol	66.7	33.3	84.8	15.2	89.5	10.5	78.9	21.1	94.7	5.3
2	Imipenem	88.9	11.1	87.9	12.1	84.2	15.8	100.0	0.0	97.4	2.6
3	Amikacin	92.6	7.4	93.9	6.1	63.2	36.8	89.5	10.5	78.9	21.1
4	Ciprofloxacin	83.3	16.7	75.8	24.2	78.9	21.1	73.7	26.3	86.8	13.2
5	Azithromycin	75.9	24.1	45.5	54.5	89.5	10.5	89.5	10.5	78.9	21.1
6	Nitrofurantoin	53.7	46.3	39.4	60.6	42.1	57.9	42.1	57.9	44.7	55.3
7	Tetracycline	72.2	27.8	84.8	15.2	36.8	63.2	68.4	31.6	52.6	47.4
8	Ceftazidime	85.2	14.8	81.8	18.2	68.4	31.6	94.7	5.3	94.7	5.3
9	Gentamicin	98.1	1.9	100.0	0.0	26.3	73.7	52.6	47.4	97.4	2.6
10	Cotrimoxazole	59.3	40.7	60.6	39.4	78.9	21.1	47.4	52.6	34.2	65.8

*S* = sensitive; *R* = resistant.

**Table 6:** Frequency of ESBL and MBL producing Gram negative isolates.

Serial number	Isolates	Total number of isolates	ESBL producers *n* (%)	MBL producers *n* (%)
1	*E. coli*	54	7 (13.0)	3 (5.6)
2	*Klebsiella* spp.	33	5 (15.1)	2 (6.1)
3	*Pseudomonas aeruginosa*	19	4 (21.0)	2 (10.5)
4	*Salmonella* spp.	19	1 (5.3)	0
5	*Enterobacter aerogenes*	38	0	0
	Total	163	17 (10.4)	7 (4.3)

## Data Availability

The data used to support the findings of this study are included within the article.
